# Supplementary material for: Cytosolic lipid droplets as engineered organelles for production and accumulation of terpenoid biomaterials in leaves
Source: Nat Commun. 2019 Feb 20;10:853. doi: 10.1038/s41467-019-08515-4 (PMC6382807; doi:10.1038/s41467-019-08515-4)
Supplement: Supplementary file 1 — Supplementary Information [file 41467_2019_8515_MOESM1_ESM.pdf]

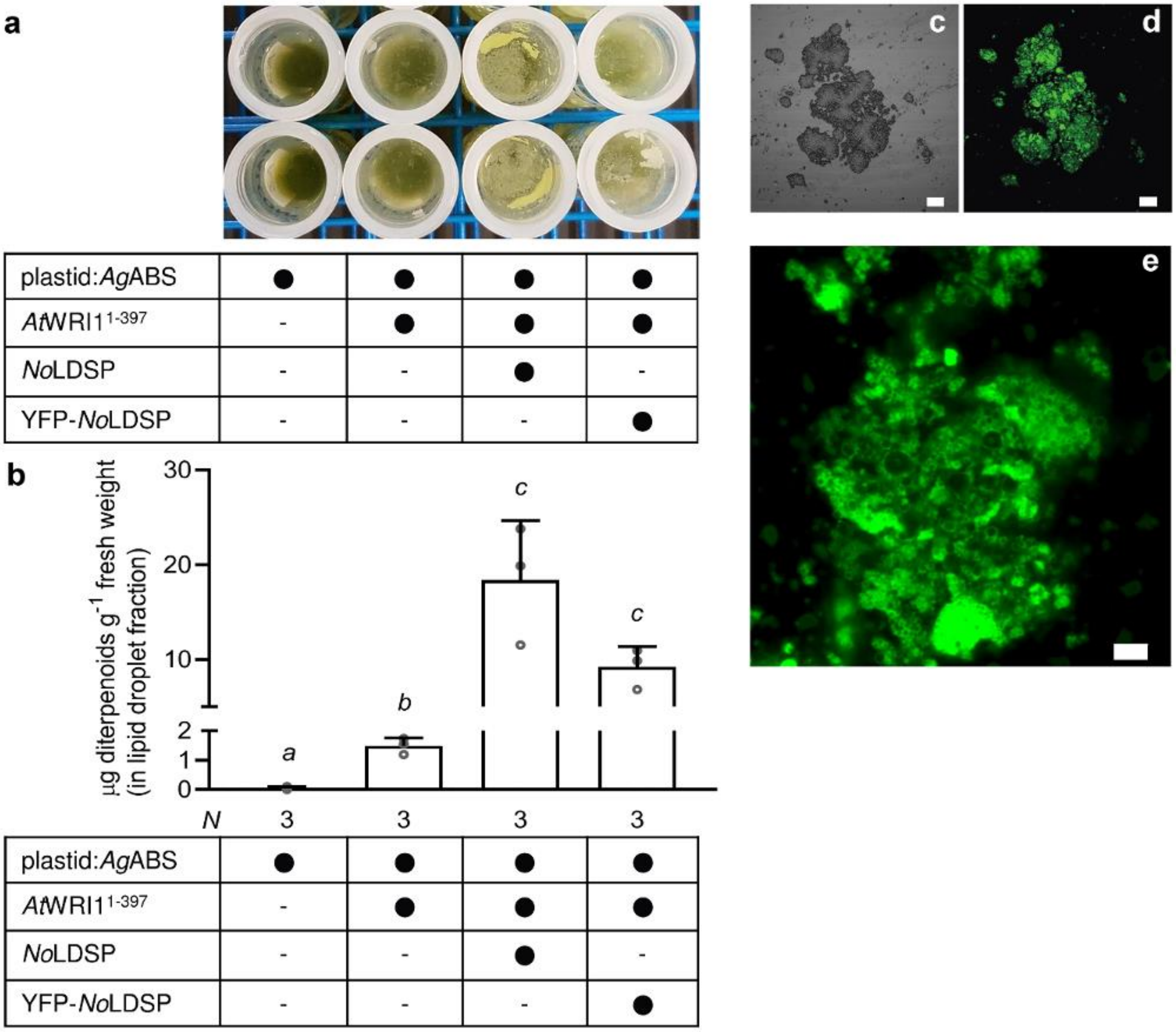

Supplementary Figure 1. Diterpenoids were sequestered in isolated lipid droplet fractions. Lipid droplet fractions were isolated from *N. benthamiana* leaves producing either plastid:*Ag*ABS alone or in combination with *At*WRI1$^{1\text{-}397}$ and *No*LDSP without or with YFP-tag. The different engineering approaches are indicated in **a** and **b** (•, was included; -, was not included). The top view of the floating lipid droplet layers after gradient centrifugation is shown (**a**). The diterpenoid content in the isolated lipid droplet fractions is given with open circles representing individual biological replicates and bars representing average values and SD (*N*=3) (**b**). Data were analyzed by Shapiro-Wilk, Brown-Forsythe ANOVA (*P* 0.0302) and Welch ANOVA (*P* 0.0041) followed by *t*-tests (unpaired, two-tailed, Welch correction, *P*<0.05). Statistically significant differences are indicated by *a-c* based on *t*-tests. *No*LDSP promotes clustering of small lipid droplets. The localization of yellow fluorescent fusion protein-tagged *No*LDSP (YFP-*No*LDSP) in clustered lipid droplets was imaged by confocal laser scanning microscopy on a collected lipid droplet fraction (**c,** phase contrast; **d**, YFP channel, scale bar 50 µm; **e**, YFP channel, scale bar 5 µm). Source Data are provided as a Source Data file.

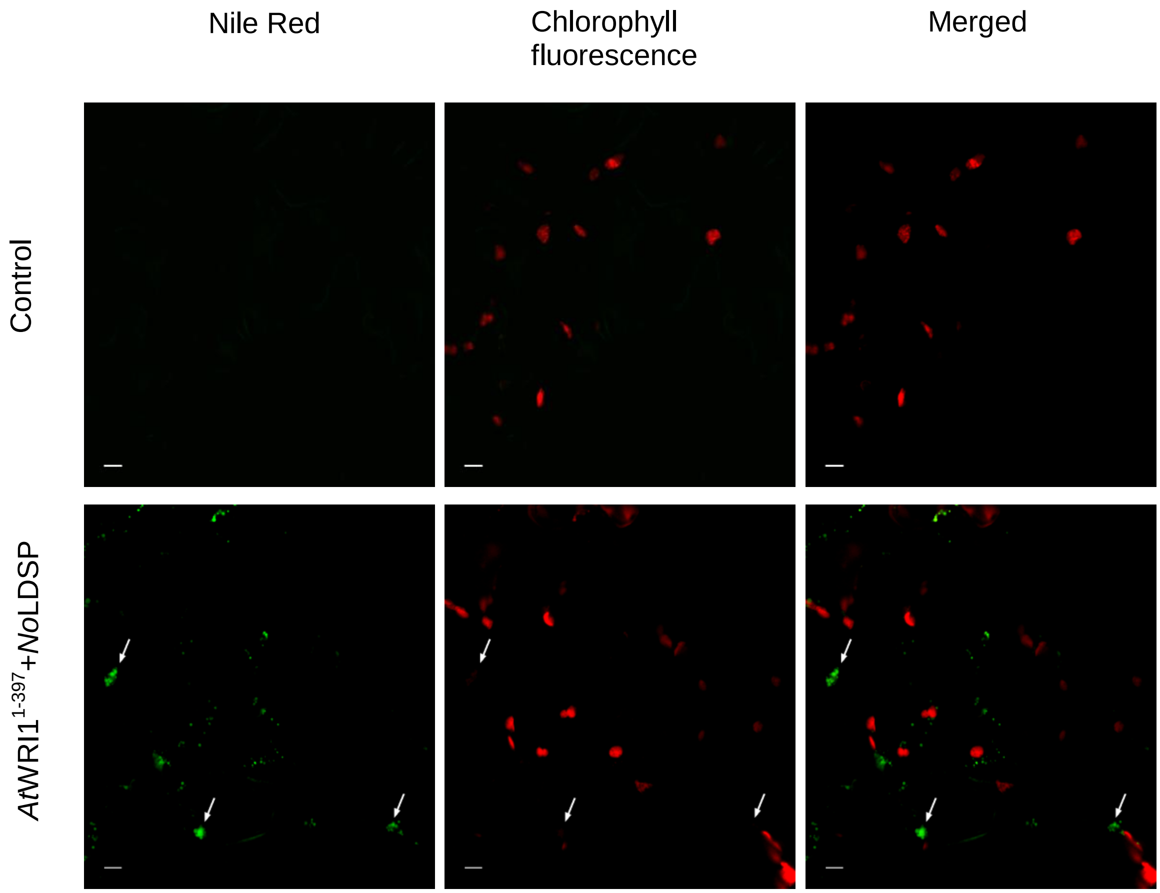

Supplementary Figure 2. *No*LDSP promotes clustering of small lipid droplets. Representative confocal laser scanning microscopy images are shown for *N. benthamiana* leaves producing either *AtWRI1*$^{1\text{-}397}$ (Control) or *AtWRI1*$^{1\text{-}397}$+*NoLDSP*. Nile red staining and confocal laser scanning microscopy were used to detect lipid droplets in mesophyll cells. Under the experimental conditions, the control accumulated a very low level of triacylglycerol in lipid droplets that was often too low to be visualized by Nile Red staining. Expression of *AtWRI1*$^{1\text{-}397}$+*NoLDSP* enhanced triacylglycerol accumulation and resulted in clustering of small lipid droplets (green) as indicated by arrows (scale bars: 5 μm).

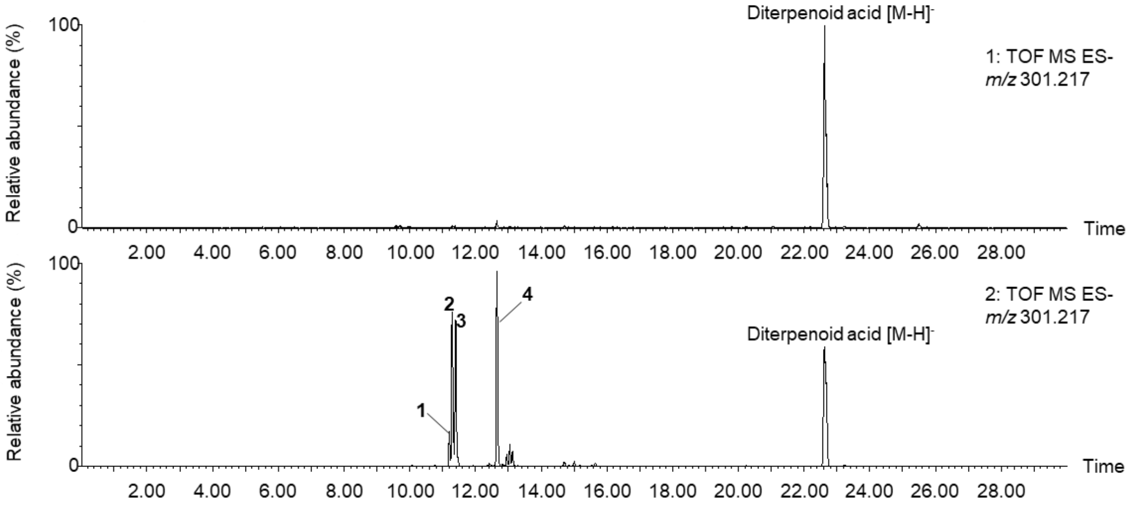

Supplementary Figure 3. LC/MS analysis of a leaf extract. The extract was prepared from a *N. benthamiana* leaf producing *At*WRI1$^{1-397}$+*No*LDSP+*El*HMGR$^{159-582}$+cytosol:*Mt*GGDPS+LD:*Ag*ABS$^{85-868}$+ER:*Ps*CYP720B4. Extracted ion chromatograms *m/z* 301.217 are shown in acquisition function 1 (0 V) and function 2 (20-80 V). Compounds **1**-**4** were subjected to MS/MS analysis. The elution order and MS/MS data were consistent with compound **1**-**3** and compound **4** being formate adducts of tetrahexosyl diterpenoid acid isomers and trihexosyl diterpenoid acid, respectively (Supplementary Figures 4 and 5).

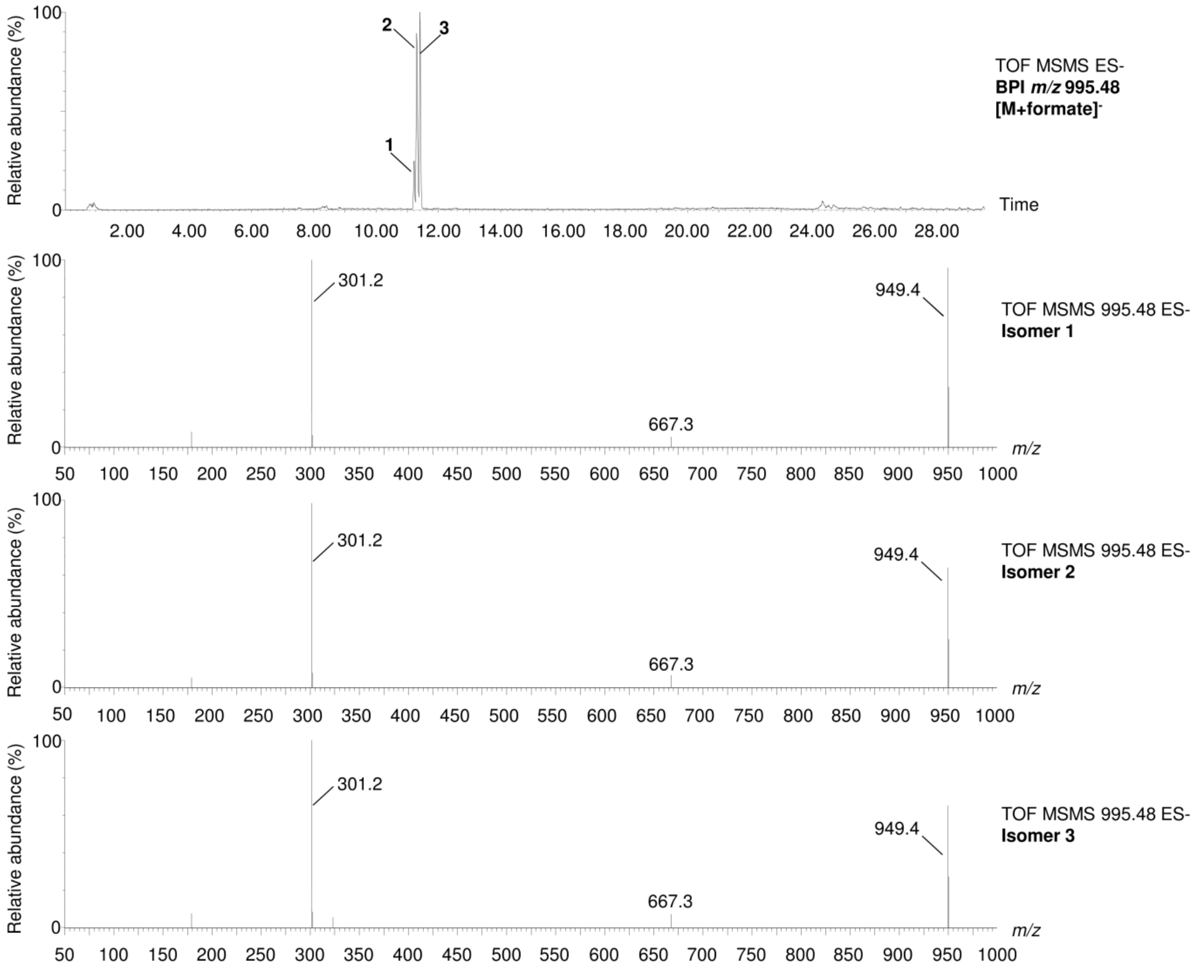

Supplementary Figure 4. LC/MS/MS analysis of tetrahexosyl diterpenoid acid isomers (1-3). An extract from a *N. benthamiana* leaf producing *At*WRI1$^{1\text{-}397}$+*No*LDSP+*El*HMGR$^{159\text{-}582}$+cytosol:*Mt*GGDPS+LD:*Ag*ABS$^{85\text{-}868}$+ER:*Ps*CYP720B4 was subjected to LC/MS/MS analysis. Accurate masses and MS/MS spectra of compounds **1-3** are consistent with formate adducts of tetrahexosyl diterpenoid acid isomers [M+formate]$^-$ *m/z* 995.4 (fragments: [M-formate]$^-$ *m/z* 949.4, [M-formate-partial loss of dihexosyl]$^-$ *m/z* 667.3 and [M-formate-tetrahexosyl]$^-$ *m/z* 301.2).

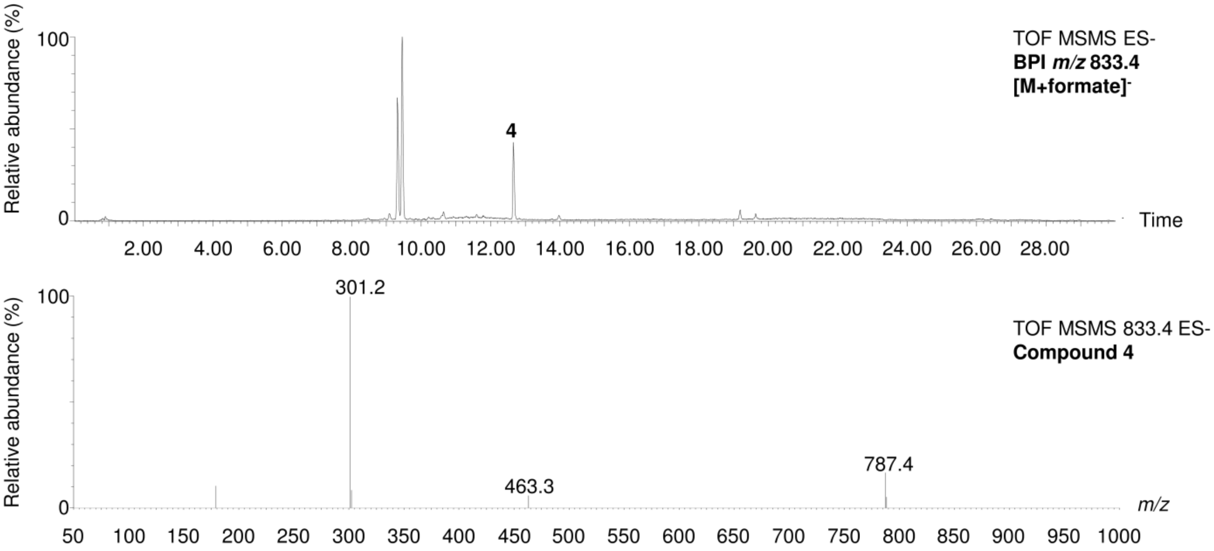

Supplementary Figure 5. LC/MS/MS analysis of a trihexosyl diterpenoid acid (4). An extract from a *N. benthamiana* leaf producing *At*WRI1$^{1\text{-}397}$+*No*LDSP+*El*HMGR$^{159\text{-}582}$+cytosol:*Mt*GGDPS+LD:*Ag*ABS$^{85\text{-}868}$+ER:*Ps*CYP720B4 was subjected to LC/MS/MS analysis. Elemental composition and MS/MS spectrum of compound 4 are consistent with a formate adduct of trihexosyl diterpenoid acid [M+formate]$^-$ *m/z* 833.3 (fragments: [M-formate]$^-$ *m/z* 787.4, [M-formate-dihexosyl]$^-$ *m/z* 463.3 and [M-formate-trihexosyl]$^-$ *m/z* 301.2).

Supplementary Table 1. Oligonucleotide primers used in the study. Sequence modifications are highlighted in blue.

Name	Sequence 5'→ 3'	Description
4b-BamHI-AtWRI1-F	**GAATGGATCCGGACA**ATGAAGAAGC	insertion of *AtWRI1*$^{1-397}$ into pENTR4
4b-XhoI-AtWRI1-R	**GAATCTCGAGTCA**CTCTCTTCCCAC	insertion of *AtWRI1*$^{1-397}$ into pENTR4
No-LDSP_F	ATGGCCGGCCCCATCATG	amplification of *NoLDSP*
No-LDSP+Stop_R	TTACTCCTTGAGGAGGAACTTCGCCAC	amplification of NoLDSP
6b-NcoI-NoLDSP_F2	**CAGTCACCATGG**CCGGCCCCATCATG	insertion of *NoLDSP* into pENTR4
6b-EcoRV-NoLDSP+Stop_R2	**CCACAGGATATC**TTACTCCTTGAGGAGGAACTTCGC	insertion of *NoLDSP* into pENTR4
ElHMGR_full_F	ATGGATTCCACTCGGCCGGAATC	amplification of full-length *ElHMGR*
ElHMGR+Stop_R	TTAAGATGATGCAAATTTGGTTACATCTTTGC	amplification of full-length *ElHMGR*
ElHMGRtruncated_infusion_F	**GCAGGCTCCACCATG**ATTTCGCCTCTGGCATC	amplification of *ElHMGR*$^{159-582}$, In-Fusion cloning into pENTR4
ElHMGR+Stop_infusion_R	**AAGCTGGGTCTAGAT**TTAAGATGATGCAAATTTGGTTAC	amplification of *ElHMGR*$^{159-582}$, In-Fusion cloning into pENTR4
At-FPS2_F	ATGGCGGATCTGAAATCAACCTTC	amplification of *cytosol:AtFDPS* from *Arabidopsis thaliana*
At-FPS+Stop_R	CTACTTCTGCCTCTTGTAGATCTTAGC	amplification of *cytosol:AtFDPS* from *Arabidopsis thaliana*
6b-EcoRV-AtFPS+Stop_R	**CCACAGGATATC**CTACTTCTGCCTCTTGTAGATCTTAGC	amplification of *cytosol:AtFDPS* from *Arabidopsis thaliana*, insertion into pENTR4
6b-NcoI-AtFPS2_F2	**CAGTCACCATGG**CGGATCTGAAATCAACCTTC	amplification of *cytosol:AtFDPS* from *Arabidopsis thaliana*, insertion into pENTR4
AtFPPS2 ptarget_F	**GGCGGAAGAGTTAAC**ATGGCGGATCTGAAATCAAC	fusion of *cytosol:AtFDPS* to the coding sequence of the plastid targeting sequence of the *Arabidopsis thaliana* ribulose bisphosphate carboxylase small chain 1A to generate *plastid:AtFDPS*, In-Fusion cloning into pENTR4
AtFPPS2 ptarget_R	**AAGCTGGGTCTAGAT**CTACTTCTGCCTCTTGTAG	amplification *plastid:AtFDPS*, In-Fusion cloning into pENTR4
CfDXS-F	ATGGCGTCTTGTGGAGCTATCGG	amplification of full-length *PbDXS* from *Plectranthus barbatus* (*Coleus forskholii*)
CfDXS-R	CATGTTGATCAAATGAAGACTGTCTTTCC	amplification of full-length *PbDXS* from *Plectranthus barbatus* (*Coleus forskholii*)
CfDXS_F	**GCAGGCTCCACC**ATGGCGTCTTGTGGAGCTATC	amplification of *Pb*DXS, In-Fusion cloning into pENTR4
CfDXS+Stop_R	**AAGCTGGGTCTAGATTCA**CATGTTGATCAAATGAAGACTG	amplification of *Pb*DXS, In-Fusion cloning into pENTR4
GGPPS1_F	ATGGCCTTCTCCGCGACATTT	amplification of full-length *ElGGDPS1* from *Euphorbia lathyris*

GGPPS1_R	TCAATTTTGCCTACTAGCAAT	amplification of full-length *ElGGDPS1* from *Euphorbia lathyris*
GGPPS1-IF-F1	**GCAGGCTCCACC**ATGGCCTTCTCCGCGACATTTTC	amplification of *ElGGDPS1*, In-Fusion cloning into pENTR4
GGPPS1-IF-R	**AAGCTGGGTCTAGAT**TCAATTTTGCCTACTAGCAATATAATTAG	amplification of *ElGGDPS1*, In-Fusion cloning into pENTR4
GGPPS2_F	ATGAACTCCATGAATTTGGGT	amplification of full-length *ElGGDPS2* from *Euphorbia lathyris*
GGPPS2_R	TCAATTCTGCCTATAAGCAAT	amplification of full-length *ElGGDPS2* from *Euphorbia lathyris*
GGPPS2-IF-F	**GCAGGCTCCACC**ATGAACTCCATGAATTTGGGT	amplification of *ElGGDPS2*, In-Fusion cloning into pENTR4
GGPPS2-IF-R	**AAGCTGGGTCTAGAT**TCAATTCTGCCTATAAGCAAT	amplification of *ElGGDPS2*, In-Fusion cloning into pENTR4
GGPPS3_F	ATGGTAGCAACTGATAAGTTT	amplification of full-length *TsGGDPS* from *Tolypothrix* sp. PCC 7601
GGPPS3_R	TTAGTGATTGCGGCTGATAAT	amplification of full-length *TsGGDPS* from *Tolypothrix* sp. PCC 7601
GGPPS3-PT-F1	**GGCGGAAGAGTTAAC**ATGGTAGCAACTGATAAGTT	fusion of *TsGGDPS* to the coding sequence of the plastid targeting sequence of the *Arabidopsis thaliana* ribulose bisphosphate carboxylase small chain 1A to generate *plastid:TsGGDPS*, In-Fusion cloning into pENTR4
GGPPS3-PT-R1	**AAGCTGGGTCTAGAT**TTAGTGATTGCGGCTGAT	amplification of *plastid:TsGGDPS*, In-Fusion cloning into pENTR4
infusion_cytMtGGPPS_F	**GCAGGCTCCACC**ATGATGGAGGTAATGGAC	amplification of *cytosol:MtGGDPS*, In-Fusion cloning into pENTR4
infusion_cytMtGGPPS_R	**AAGCTGGGTCTAGAT**TTAGTGTTCACGCTCTAACAC	amplification of *cytosol:MtGGDPS*, In-Fusion cloning into pENTR4
PcPatS_1F	ATGGAGTTGTATGCCCAAAGTGTTGGAG	amplification *cytosol:PcPAS* (full-length *PcPAS*) from *Pogostemon cablin*
PcPatS_1R	TTAATATGGAACAGGGTGAAGGTACAACTGC	amplification *cytosol:PcPAS* (full-length *PcPAS*) from *Pogostemon cablin*
PcPatS_2F	**GCAGGCTCCACC**ATGGAGTTGTATGCCCAAAGTGTT	amplification *cytosol:PcPAS*, In-Fusion cloning into pENTR4
PcPatS ptarget_F	**GGCGGAAGAGTTAAC**ATGGAGTTGTATGCCCAAAG	fusion of *cytosol:PcPAS* to the coding sequence of the plastid targeting sequence of the *Arabidopsis thaliana* ribulose bisphosphate carboxylase small chain 1A to generate *plastid:PcPAS*, In-Fusion cloning into pENTR4
PcPatS ptarget_R	**AAGCTGGGTCTAGAT**TTAATATGGAACAGGGTG	amplification of *cytosol:PcPAS* and *plastid:PcPAS*, In-Fusion cloning into pENTR4
Ag_AbS_1F	ATGGCCATGCCTTCCTCTTCA	amplification of *plastid:AgABS* (full-

		length *AgABS)* from *Abies grandis*
Ag_AbS_1R	TTAGGCAACTGGTTGGAAGAGGCA	amplification of *plastid:AgABS* (full-length *AgABS)* from *Abies grandis*
AgAbS_2F	**GCAGGCTCCACC**ATGGCCATGCCTTCCTCTTCATTG	amplification of *plastid:AgABS*, In-Fusion cloning into pENTR4
infusion_AgAbS_F1	**GCAGGCTCCACCATG**GTGAAACGAGAATTTCCT	amplification of *cytosol:AgABS*$^{85\text{-}868}$, In-Fusion cloning into pENTR4
infusion_AgAbS+Stop_R1	**AAGCTGGGTCTAGAT**TTAGGCAACTGGTTGGAAG	amplification of plastid:AgABS and *cytosol:AgABS*$^{85\text{-}868}$, In-Fusion cloning into pENTR4
infusion_AgAbs LDSP_R3	**GATGGGGCCGGCCAT**GGCAACTGGTTGGAAGAG	fusion of *cytosol:AgABS*$^{85\text{-}868}$ to *NoLDSP* to generate *LD:AgABS*$^{85\text{-}868}$, In-Fusion cloning into pENTR4
infusion_CYP720B4_F2	**GCAGGCTCCACC**ATGGCGCCCATGGCAGACCAAATATC	amplification of *ER:PsCYP720B4* (full-length *PsCYP720B4*), In-fusion cloning into pENTR4
infusion_CYP720B4_F3	**GCAGGCTCCACCATG**AATATCCAGAGAGGCCCAAAAATG	amplification of *cytosol:PsCYP720B4*$^{30\text{-}483}$, In-fusion cloning into pENTR4
infusion_CYP720B4_F1	**TTCCTCCTCAAGGAG**AATATCCAGAGAGGCCCA	fusion of *cytosol:PsCYP720B4*$^{30\text{-}483}$ to *NoLDSP* to generate *LD:PsCYP720B4*$^{30\text{-}483}$
infusion_CYP720B4_R1	**AAGCTGGGTCTAGAT**TTATTCATTCTCTACTCTACCATG	amplification of *ER:PsCYP720B4*, *cytosol:PsCYP720B4*$^{30\text{-}483}$ and *LD:PsCYP720B4*$^{30\text{-}483}$, In-Fusion cloning into pENTR4
infusion_CaCPR_F1	**TTCCTCCTCAAGGAG**TCGTCAGGAAAGTCGGGGA	fusion of *cytosol:CaCPR*$^{70\text{-}708}$ to *NoLDSP* to generate *LD:CaCPR*$^{70\text{-}708}$, In-Fusion cloning into pENTR4
infusion_CaCPR_R1	**AAGCTGGGTCTAGAT**TCACCACACATCACGCAAATACCT	amplification of *LD:CaCPR*$^{70\text{-}708}$, In-Fusion cloning into pENTR4
