# Supplementary material for: Cytosolic lipid droplets as engineered organelles for production and accumulation of terpenoid biomaterials in leaves
Source: Nat Commun. 2019 Feb 20;10:853. doi: 10.1038/s41467-019-08515-4 (PMC6382807; doi:10.1038/s41467-019-08515-4)
Supplement: Supplementary file 3 — Description of Additional Supplementary Files [file 41467_2019_8515_MOESM3_ESM.pdf]

Description of Additional Supplementary Files

File Name: Supplementary Data 1
Description: Codon optimized sequences
